# Applicability of Isolates and Fractions of Plant Extracts in Murine Models in Type II Diabetes: A Systematic Review

**DOI:** 10.1155/2016/3537163

**Published:** 2016-10-24

**Authors:** Gabriela Diniz Pinto Coelho, Vanessa Soares Martins, Laura Vieira do Amaral, Rômulo Dias Novaes, Mariáurea Matias Sarandy, Reggiani Vilela Gonçalves

**Affiliations:** ^1^Department of Medicine and Nursing, Federal University of Viçosa, Viçosa, MG, Brazil; ^2^Institute of Biomedical Science, Department of Structural Biology, Federal University of Alfenas, Alfenas, MG, Brazil; ^3^Department of General Biology, Federal University of Viçosa, Viçosa, MG, Brazil; ^4^Department of Animal Biology, Federal University of Viçosa, MG, Brazil

## Abstract

Type II diabetes mellitus is one of the most common public health problems worldwide. Its increasing prevalence in several countries and the difficult metabolic control of individuals with the disease justify studying strategies for primary prevention. The population has sought alternative and cheaper ways to treat the disease, including the use of plants considered medicinal by the population. In this study, we carried out a systematic review on the applicability of isolates and fractions of plant extracts in animal models in type II diabetes. A literature search was performed in MEDLINE/PubMed and Scopus databases. Studies using other experimental animals (horses, rabbits, and monkeys) and humans as well as articles in Chinese, German, and Russian were excluded. We assessed the quality of the studies included by using the criteria described in the ARRIVE guidelines. In general, the animals that received fractions or isolates presented reduced blood glucose levels, normalization of body weight and plasma insulin levels, and reduced total triglycerides and cholesterol. In addition, we observed wide variation among the analyzed parameters, which hindered comparison between the studies found. In further studies, standardized reports and experimental design would help to establish comparable study groups and advance the overall knowledge, thus facilitating translatability from animal data to human clinical conditions.

## 1. Introduction

Diabetes mellitus (DM) is a syndrome caused by changes in the metabolism of carbohydrates, lipids, and proteins and may occur in two different forms: type I (10%) and type II (90%) [1]. Type I diabetes results from body inability to produce insulin [2], while type II may be caused by a failure in the production and secretion of insulin by the pancreas, due to insufficient production or a problem in the beta-cell receptors, thus decreasing the sensitivity of the target tissue to the metabolic effect of this hormone. This decreasing sensitivity is known as insulin resistance [3]. Patients with this disease present polydipsia (excessive thirst), polyuria (urine production over 2.5 liters/day), polyphagia (excessive avidity for food), and delayed wound healing [4]. Besides, poor glucose metabolism, reduced insulin signaling, excessive release of free fatty acids, and interleukin-6 are changes also considered important for analysis in clinical and preclinical studies [5].

According to the World Health Organization (2012), diabetes mellitus accounts for 3.5% of the noncommunicable diseases in the world. The International Diabetes Federation (IDF) [6] estimates that, by 2013, more than 382 million people around the world had been affected by the disease, and the figures are increasing in all countries. Portugal, for example, is the second country in Europe with the highest prevalence of diabetes mellitus: 12.7 people with DM per 100 inhabitants in 2011 [7].

In Brazil, according to estimates, there are more than 12 million people with type II diabetes mellitus (DM2) [8], mainly people who are over 40 years of age and obese. However, recent studies have shown a considerable increase in the number of children and teenagers with the disease, which may be associated with bad eating habits and lack of physical activity. This leads to increasing rates of obesity, which is considered a risk condition for the development of type II diabetes [9].

The population has sought alternative and cheaper treatments for the disease, including the use of plants considered medicinal by the population. In several countries, such as Japan, China, and India, the use of medicinal plants and their derivatives is increasing, since they are considered simple, cheap, and effective treatment alternatives [10, 11]. There are some reports in the literature about the benefits of different herbal treatments on several metabolic changes caused by type II diabetes. The studies generally show positive effects of plant extracts on the normalization of body weight and decrease of glucose levels, total cholesterol, and triglycerides [12].

Therefore, the use of phytotherapy has opened a new perspective for the management and treatment of metabolic diseases, such type II diabetes, since it is an affordable treatment [13]. However, the use of most of these plants has not been investigated [14, 15]. Research on plant extracts should be conducted to ensure that they are effective and safe for the population [16]. Due to these factors and the high number of type II diabetes carriers, the demand for less expensive therapies may significantly benefit population health [11].

*In vivo* and* in vitro* studies are commonly used in biomonitoring research of plant extracts, aiming to identify their biological activity. The fractions are metabolites obtained by fractioning plant extracts, which provides a more specific analysis of the plant active principle [17]. These studies are important for the treatment of chronic diseases, including diabetes, since the treatment or control of these diseases is expensive and the number of affected people has increased considerably. Besides the fractions, the isolates obtained from plants have also played an important role in the treatment of metabolic disorders. After isolating a particular component of the crude extract, it is possible to ensure that the effects on the tissue are caused only by a specific constituent of the extract. This makes the molecule more attractive to the drug market, which aims to develop drugs from plants or other materials.

A systematic review is based on predetermined criteria and consistent scientific evidence. It aims to collaborate with research selection and/or tools for the development of products based on original information [18], with well-defined criteria selection, to ensure the quality of the summarized studies and their reproducibility. Moreover, a conclusion providing new information based on filtered content is necessary [19]. Generally, due to its rigorous methods to identify, select, collect, and analyze data, this kind of study provides the highest level of scientific evidence. Therefore, our study aimed to make a descriptive and critical analysis of studies on the activity of plant fractions and isolates in the treatment of type II diabetes in animal models.

## 2. Material and Methods

### 2.1. Selection of Papers

The papers analyzed in this review were selected from two electronic databases, PubMed and Scopus, accessed on September 3, 2015, using the search filters: “animal model”, “plant extract”, and “diabetes mellitus type II”. These filters have been developed for the search on PubMed, according to the Medical Subject Headings (MeSH terms), used for a more efficient indexing of publications on the subject under study [20]. In order to expand the search, MeSH terms were combined with the title and abstract (TIAB). A standard filter was used [21] to identify all studies with animals in PubMed. The terms used to search on PubMed were adapted for the selection of Scopus publications, and the “animal model” filter was provided by the site itself (Supplemental Data 1 in Supplementary Material available online at http://dx.doi.org/10.1155/2016/3537163).

The Prism guideline was used to develop this review [22]. After the papers were collected from the two electronic databases, the duplicates were excluded by comparing the title, author, year, and country. A screening was performed for title and abstract, guided by the eligibility criteria:* in vivo* experimental studies; studies using rats or diabetic mice; use of fractions or isolates of noncommercial plants; treatment of the main symptoms of type II diabetes; studies written in English or Portuguese.

**Figure 1:**
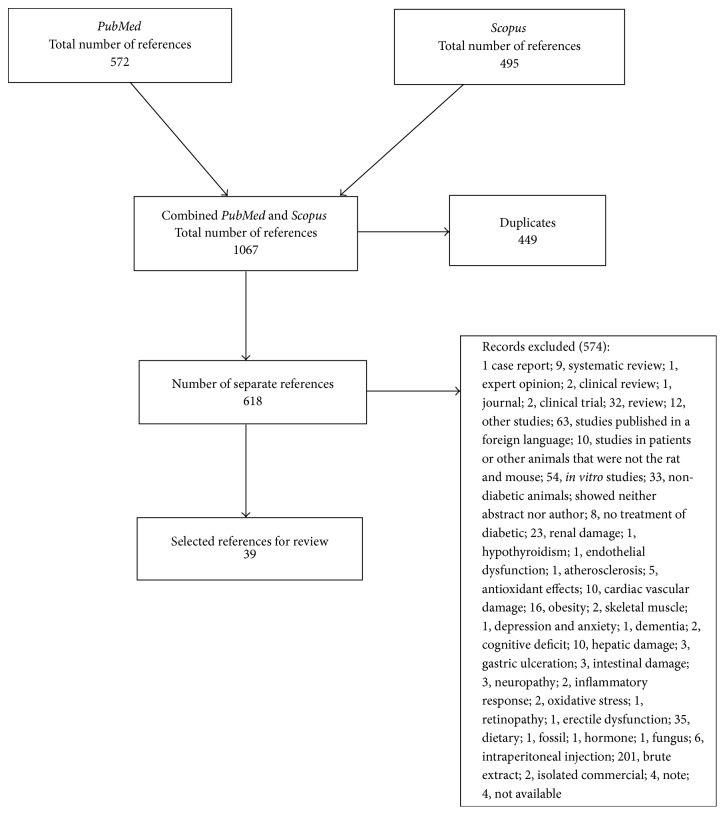
Results obtained after the advanced search in the databases. After literature review, 39 papers were selected from Moher D, Liberati A, Tetzlaff J, Altman DG, and the PRISMA Group (2009). Preferred reporting items for systematic reviews and meta-analyses: the PRISMA Statement. PLoS Med 6(6): e1000097. doi: 10.1371/journal.pmed1000097. For more information, visit www.prisma-statement.org. Prism: systematic review.

Next, all selected papers were obtained in full for a second screening, when all of them were examined to select those that met the criteria for the inclusion in the systematic review. Those unavailable on the internet were requested from their respective authors. When they did not respond, the studies were excluded. The entire search process, exclusion, and the number of selected papers were described in detail in the PRISMA Guideline (Figure 1).

### 2.2. Qualitative Characteristics of Publications

After screening, the papers were reviewed.  Table 2 shows the description of the main characteristics of the studies. The following parameters were assessed: (1) publication features: author, year, and country; (2) experimental features: animal model, species, sample number, sex, weight, age, type of caging used, number of animals per cage, number of experimental groups and number of animals in each group, if randomization was made, and control groups; (3) treatment features: plant species used, name of the fraction or isolate, dose, route of administration, and treatment duration; (4) diabetes induction: drug used, dose, route of administration, and testing to prove diabetes occurrence (Table 1).

### 2.3. ARRIVE (Bias Analyses)

The detailed reports of experiments are crucial in the review process, so that they can be validated and used as a source of information for further research. However, many studies do not bring relevant or concise information, which leads to the realization of redundant and duplicated experiments [23]. Therefore, guidelines were developed for animal research reports, such as the ARRIVE guideline, based on the CONSORT Statement. The ARRIVE guidance is a list of 20 items that describe the minimum information that all scientific publications reporting research using animals must include, aiming at high quality reports and critical and accurate review of what was performed and found [24]. Thus, based on these fundamentals and the objective of this study, a table displaying the most relevant and applicable items from the ARRIVE was developed for a critical evaluation of the studies included in this review (Table 2). The authors assessed the quality, integrity, and transparency of each publication. Divergent opinions were resolved by consensus.

## 3. Results

### 3.1. Prism

The search conducted in this study found a total of 1,067 papers, out of which 571 were found in PubMed and 495 in Scopus. Out of this total, 449 papers were duplicates; thus 618 studies remained. Then, a title and abstract screening was performed, guided by the eligibility criteria listed above. In this respect, 574 studies were excluded due to inadequate research topic. Among the excluded studies, we can highlight those on the crude extract of the plant (200), studies in languages other than English and Portuguese (63), secondary studies, literature reviews, editorials, comments (60),* in vitro* studies (54), and studies in which alcohol was administered in the diet (35). Next, 39 studies were selected and their reference lists were screened to identify additional relevant studies missed in the initial search strategy. Thus, all the studies that met the eligibility criteria were included in the review, taking into account the use of fractions and isolates from noncommercial plants in the treatment of type II diabetes in animal models of rats and mice. All search process is shown in Figure 1.

### 3.2. Qualitative Results

**Figure 2:**
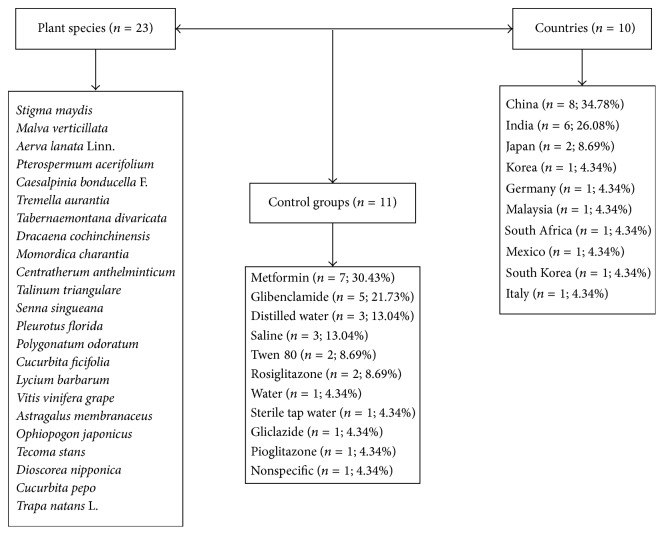
Summary of the articles describing the main fractions of plants, their species, families, used control groups, and the main countries where researches on this topic have been developed. Data obtained from the qualitative and ethnobotanic analysis. Flowchart fractions.

With respect to papers reporting treatments with plant fractions (*n* = 23), the years of publication ranged from 2001 to 2015. Most studies used rats (60.9%) and mice (39.1%). The sample size varied greatly; some studies used 14 animals and others, 98 animals, while 36.4% of the publications did not report such data. Most studies used male animals, but 2 papers reported the use of both sexes, and 21.7% of the studies did not provide this information. The age of the animals ranged from 3 to 14 weeks and 56.5% of the studies did not report these data. The weight of the animals was not reported in 21.74% of the studies. Only 26.1% of the papers reported if randomization was applied in the experimental groups. Fractions of the extracts were administered orally in 91.29% of the studies and the treatment duration ranged from 7 days to 10 weeks. Regarding the drug used to induce type II diabetes, 60.86% of the studies used streptozotocin; 8.69%, alloxan; and 4.35%, PX-407 (Table 1). China (34.9%) and India (26%) are the countries with the largest number of publications on this subject. Around 47.8% of the studies used a control group. Metformin (30.5%) and glibenclamide (21.7%) were the most commonly used drugs (Figure 2).

**Figure 3:**
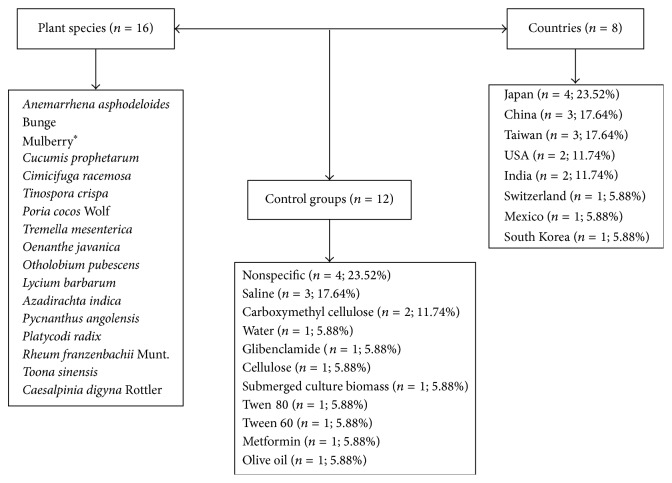
Summary of the articles describing the main isolates of plants, their species, families, used control groups, and the main countries where a research on this topic has been developed. Data obtained from the qualitative and ethnobotanic analysis. Flowchart isolates. ^*∗*^The popular name of the plant because the scientific name was not found.

The studies that used plant isolates in the treatment of diabetes (*n* = 16) were carried out from 1998 to 2014. Mice (56.25%), rats (37.50%), and both (6.25%) were the species used in the experiments. The sample size ranged from 12 to 102 animals, and 37.50% of the studies did not report such information. Most studies used male animals (87.50%) and 12.50% used both sexes. The age of the animals ranged from 7 to 26 weeks. The weight of the animals was not reported in 75% of the papers and the strains used were not provided in 31.25% of the studies, while 62.50% of the papers did not report animal randomization. The treatment was administered orally in all analyzed studies (100%). Diabetes was induced with the use of streptozotocin (56.3% of the studies), PEG300 (6.25%), and insulin solution (12.5%) (Table 2). Japan (23.5%) stands out among the countries that have developed studies in the area, followed by India and Taiwan (17.64%) (Figure 3).

**Figure 4:**
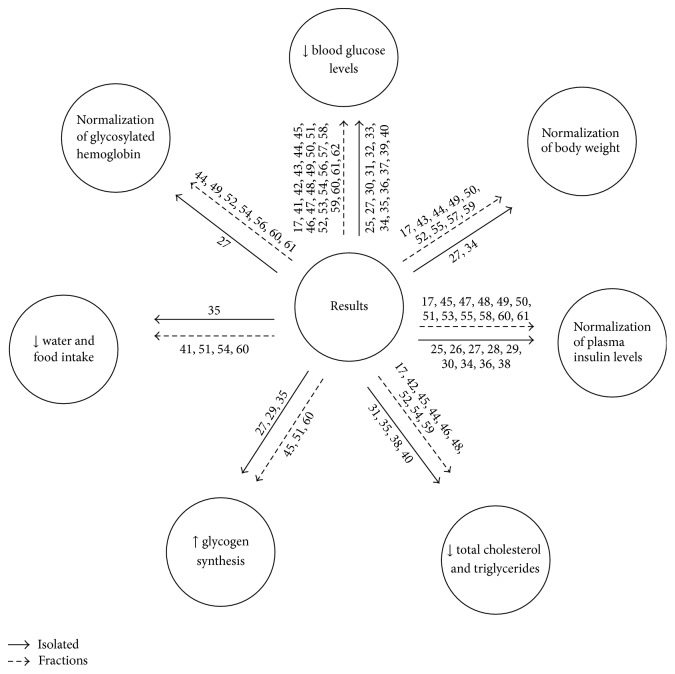
Main results demonstrating the action of fractions and isolates from plants on the treatment of type II diabetes. Flowchart: isolates and fractions.

The main results for plant fractions and isolates in the treatment of type II diabetes are shown in Figure 4. The main findings were (A) reduced blood glucose levels in isolate treatments (25, 27, 30, 31, 32, 33, 34, 35, 36, 37, 39, and 40) and fractions (17, 41, 42, 43, 44, 45, 46, 47, 48, 49, 50, 51, 52, 53, 54, 56, 57, 58, 59, 60, 61, and 62); (B) normalization of body weight in studies using fractions (17, 43, 44, 49, 50, 52, 55, 57, and 59) while only two studies evaluated this parameter in isolates (27 and 34); (C) normalization of plasma insulin levels during treatment with fractions (17, 45, 47, 48, 49, 50, 51, 53, 55, 58, 60, and 61) and treatment with isolates (25, 26, 27, 28, 29, 30, 34, 36, and 38); (D) reduced total triglycerides and cholesterol in studies using fractions (17, 42, 45, 44, 46, 48, 52, 54, and 59) and isolates (31, 35, 38, and 40); (E) increased glycogen synthesis in studies using fractions (45, 51, and 60) and isolates (27, 29, and 35). Decreased water and food intake has been described in only one isolate (35) and four fractions (41, 51, 54, and 60). The normalization of glycosylated hemoglobin was observed in seven fractions (44, 49, 52, 54, 56, 60, and 61) and one isolate (27) (Figure 4).

### 3.3. ARRIVE (Bias Analysis)

The ARRIVE guidelines were used to assess the quality of the papers under analysis (Table 1). After reading and performing critical analyses, the researchers observed that 84.61% of the studies had exact title and concise description. Abstracts describing the purpose, methods, main results, and conclusions were found in 92.30% of the studies. Primary and secondary objectives were clearly stated by 82.05% of the studies, while 92.30% reported in the methodology description that they had obtained permission from the ethics committee for performing the research; on the other hand, experimental information about controlled or blind study was observed in only 15.38%. The animal species were cited in 89.74% of the papers, while weight and sex were described in only 56.41% of the studies. It was observed that 46.15% of the publications reported genetic changes in animals, while lodgment and environmental conditions (light/dark cycle, temperature, and water) were reported in 35.89% and 84.61% of the studies, respectively. Regarding the sample size, 56,41% reported the total number of used animals, but only 5.12% explained the reason for choosing such numbers, and 28.20% of the authors reported the use of randomization. It was observed that 87.17% of the studies specified each statistical analysis method. Only 7.69% of the papers reported the occurrence of animal mortality during the experiment. Among the evaluated discussions, 89.74% interpreted the results taking into account the objectives and hypotheses of the study, current theory, and relevant publications. Only 30.76% commented about the limitations of the studies. Comments on the importance of applying the results to human biology were found in 56.41% of the studies.

## 4. Discussion

This review aimed to describe the main findings in literature on the effects of fractions and isolates obtained from plant extracts on the treatment of type II diabetes in murine models. We believe that the information obtained may help and provide guidance to researchers about the best animal models, drugs, and most used doses in disease induction. Besides, it will guide further research on the most common and important parameters to describe the best results for controlling the metabolic changes caused by the disease. Studies that tested crude plant extracts were not included in this review, due to their wide variability. Studies that obtained fractions and isolates commercially were also excluded. Although species differences prevent the direct extrapolation to clinical applications in humans, the current findings strongly point to the need for a more controlled preclinical research in animals and then in humans, mainly in relation to the doses of fractions and isolates and the most used plant species.

The present review showed that isolates or fractions of plants had positive effects on diabetes treatment and reduced various animal blood and tissue parameters that had been changed by the disease. This study also highlights important issues related to the quality of the models and protocols, drugs and doses used in the study for inducing the disease, the most commonly used administration routes, and main tests used for disorder confirmation. Systematic review studies are focused on the assessment of the quality of the reviewed studies, using acknowledged scales and protocols. Although these scales have not been formally developed for experimental model studies, the assessment of the quality of the reviewed studies considered the items normally included in scales for randomized clinical studies. Therefore, we used the ARRIVE platform for work quality analysis and observed that most studies did not provide many details about the materials and methods used, which prevents the replication of some studies. There were no reports of the number of animals used, age, weight, and even the presence of randomization to reduce bias in the selection of the animals and assessment of the results in many studies. These findings corroborate the need for guidelines to describe the required information for all scientific publications that use animals as experimental models [24]. In this study, out of the 618 articles analyzed, 39 were selected according to the eligibility criteria and the proposed objective. The PRISMA recommendations were used to guide the development of this systematic review and improve the visualization of the steps of an effective search [22].

Most animals studied were male, since males suffer less hormonal fluctuation and hence less change in behavior compared to females [62]. The number of studies with rats (*n* = 20) and mice (*n* = 19) was very close. However, is it possible to detect the increasing use of mice in preclinical experiments, due to the genetic similarities between this species and humans. According to Machado and Zatti [63], about 99% of human genes have been mapped in mouse, which allows the association between them. Moreover, it must be taken into account that the small size of these animals reduces the costs of the experiment and makes it easy to handle and perform a great number of procedures. There was wide variation in the age of the animals. The youngest animals were 3 weeks old and the oldest, 26 weeks. In addition, many studies did not provide such information (*n* = 18). The weight of the animals ranged on average from 25.5 g in mice to 61 g in rats. The authors attribute this great variability to the discrepancies in the age of the animals. Besides, the variable weight was not reported in 38.4% of the studies. The number of studies that did not describe variables such as age and weight is worrying, since these characteristics are important for further replication of the studies and elaboration of extensive reports on the procedures adopted [64].

Streptozotocin, either combined or not with another drug, was the main drug selected for type II diabetes induction in animals. Streptozotocin is a large spectrum antibiotic, used as a diabetogenic agent in experimental animals [65]. This action is mediated by the destruction of beta cells in the pancreas, which leads to insulin deficiency and also occurs in human type II diabetes in relation to metabolic characteristics [66]. Wide variation was observed in the streptozotocin dose used to induce diabetes, from 20 mg/kg to 137 mg/kg. The analysis of works with drug-induced diabetes mainly requires the establishment of the most appropriate dose and the correct administration time, since these variables may reduce the time and costs of the experiment. In this review, after the analysis of the work, no consensus was found for the best dose and timing for drug application. Metformin was the main drug selected for the control group, due to its relevant clinical use, favorable toxicity profile, and safety. Besides, it is well tolerated during treatment [67].

Although many drugs have been used for diabetes treatment, some of them are expensive and inefficient and cause severe side effects. Thus, there is a growing interest from researchers and pharmaceutical companies in the development of alternative drugs, such as medicinal plants, for diabetes treatment [68–70]. However, further studies should be conducted, since some plants associated with diabetes mellitus treatment are considered toxic and may cause various tissue lesions [71]. The present study reports several plant species that have been used to obtain both fractions and isolates. Most studies were carried out in China, India, and Japan. These countries have shown great interest in the development of drugs from plant extracts, due to their great flora diversity. Japan has always had an interest in the development of new technologies. Phytotherapy is promising for health care in many ways. The analysis of the results of the studies revealed that most authors reported decreased blood glucose in treated diabetic animals (*n* = 33) and normalized plasma insulin levels (*n* = 21). Postprandial hyperglycemia is a common pathogenesis of type II diabetes induced by insulin resistance, as well as the partial destruction of pancreas *β* cells [72–75]. The effective control of blood glucose and insulin level is a key step in preventing or reversing diabetic complications and improving the life quality of patients [76]. Hyperlipidemia is another complication caused by diabetes, characterized by high cholesterol and triglyceride levels and lipoprotein composition changes [77]. These data were analyzed in this study, due to their relevance. Thirteen studies reported decreased total cholesterol and triglyceride levels in animals treated with plant derived medicine.

Besides, polydipsia, polyphagia, and changes in weight (weight loss or gain) are common occurrences in patients with diabetes [78]. Weight loss is usually observed when the disease is acquired and can be associated with dehydration and catabolism of fat tissue or protein degradation and consequent muscle mass loss. According to this review, many studies reported increased animal water and food intake, as well as weight normalization. These data can be justified by the increased glucose and insulin uptake and decreased secretion of blood glucose, which indicate improved animal glycemic control [49]. These improved results in the body weight of diabetic animals are consistent and were reported by some studies using medicinal plants with potential antidiabetic effects [79, 80]. The increased glycogen synthesis was also analyzed, since the liver metabolism of this substance regulates glucose blood level [50]. In addition, some studies reported the normalization of glycated hemoglobin levels, which is important to assess diabetes control levels, since its dosage directly reflects the average blood glucose levels, from two to three months prior to the collection of the biological material [81].

## 5. Conclusion

The results of this study demonstrate that plant fractions and isolates improve the main physiological and morphological changes caused by type II diabetes and decrease food and water intake, total cholesterol, triglycerides, and glucose, thus normalizing body weight and blood insulin levels. However, serious methodological problems were found in many studies, including errors in the details of the procedures performed, which prevents the understanding of some studies and hinders the use of the data found in animals for studies on human clinical condition. Therefore, the improvements in research reports on preclinical studies require a collective effort from authors, journal editors, reviewers, and funding agencies to ensure that the papers will allow other researchers to reproduce the study.

## Supplementary Material

Descriptors used for advanced search in PubMed and Scopus.

## Figures and Tables

**(a):** 

	Title	Author/publication year	Country
Isolates	[25] A Polysaccharide Extract of Mulberry Leaf Ameliorates Hepatic Glucose Metabolism and Insulin Signaling In Rats with Type 2 Diabetes Induced by High Fat-Diet and Streptozotocin	Ren et al. (2015)	China
	[26] Antidiabetic Activity of a Xanthone Compound, Mangiferin	Miura et al. (2001)	Japan
	[27] Anti-Diabetic Effect of a Novel N-Trisaccharide Isolated from *Cucumis prophetarum *on Streptozotocin-Nicotinamide Induced Type 2 Diabetic Rats	Kavishankar and Lakshmidevi (2014)	India
	[28] Antidiabetic Effects of the *Cimicifuga racemosa* Extract Ze 450 *In Vitro* and *In Vivo* in ob/ob Mice	Moser et al. (2014)	Switzerland
	[29] Borapetoside C from *Tinospora crispa* Improves Insulin Sensitivity in Diabetic Mice	Ruan et al. (2012)	Taiwan
	[30] Dehydrotrametenolic Acid Induces Preadipocyte Differentiation and Sensitizes Animal Models of Noninsulin-Dependent Diabetes Mellitus to Insulin	Sato et al. (2002)	Japan
	[31] Effects of Ingested Fruiting Bodies, Submerged Culture Biomass, and Acidic Polysaccharide Glucuronoxylomannan of *Tremella mesenterica* Retz.:Fr. on Glycemic Responses in Normal and Diabetic Rats	Lo et al. (2006)	Taiwan
	[32] Inhibition of Glycogen Synthase Kinase-3*β* by Falcarindiol Isolated from Japanese Parsley (*Oenanthe javanica*)	Yoshida et al. (2013)	Japan
	[33] Isolation and Antihyperglycemic Activity of Bakuchiol from *Otholobium pubescens* (Fabaceae), a Peruvian Medicinal Pant Used for the Treatment of Diabetes	Krenisky et al. (1999)	USA
	[34] LBP-4a Improves Insulin Resistance via Translocation and Activation of GLUT4 in OLETF Rats	Zhao et al. (2014)	China
	[35] Meliacinolin: A Potent Α-Glucosidase and Α-Amylase Inhibitor Isolated from *Azadirachta indica* Leaves and In Vivo Antidiabetic Property in Streptozotocin-Nicotinamide-Induced Type 2 Diabetes in Mice	Perez-Gutierrez and Damian-Guzman (2012)	Mexico
	[36] Novel Terpenoid-Type Quinones Isolated from *Pycnanthus angolensis* of Potential Utility in the Treatment of Type 2 Diabetes	Luo et al. (1999)	USA
	[37] Platyconic Acid, A Saponin from *Platycodi radix*, Improves Glucose Homeostasis by Enhancing Insulin Sensitivity *In Vitro* and *In Vivo*	Kwon et al. (2012)	South Korea
	[38] Rhaponticin from rhubarb Rhizomes Alleviates Liver Steatosis and Improves Blood Glucose and Lipid Profiles in KK/Ay Diabetic Mice	Chen et al. (2009)	China
	[39] Rutin Potentiates Insulin Receptor Kinase to Enhance Insulin-Dependent Glucose Transporter 4 Translocation	Hsu et al. (2014)	Taiwan
	[40] Type 2 Antidiabetic Activity of Bergenin from the Roots of *Caesalpinia digyna *Rottler	Kumar et al. (2012)	India

Fractions	[41] A Study on Hypoglycaemic Health Care Function of *Stigma maydis* Polysaccharides	Zhang et al. (2013)	China
	[1] Antidiabetic Activities of Extract from *Malva verticillata *Seed via the Activation of AMP-Activated Protein Kinase	Jeong and Song (2011)	Korea
	[42] Antidiabetic Activity of Alkaloids of *Aerva lanata* Roots on Streptozotocin-Nicotinamide Induced Type-II Diabetes in Rats	Agrawal et al. (2013)	India
	[43] Antidiabetic Activity of *Pterospermum acerifolium* Flowers and Glucose Uptake Potential of Bioactive Fraction in L6 Muscle Cell Lines with Its HPLC Fingerprint	Paramaguru et al. (2014)	India
	[44] Antidiabetic Activity of *Caesalpinia bonducella* F. in Chronic Type 2 Diabetic Model in Long-Evans Rats and Evaluation of Insulin Secretagogue Property of Its Fractions on Isolated Islets	Chakrabarti et al. (2005)	India
	[45] Antidiabetic Effect of an Acidic Polysaccharide (TAP) from *Tremella aurantia *and Its Degradation Product (TAP-H)	Kiho et al. (2001)	Japan
	[46] Antidiabetic Effect of Orally Administered Conophylline-Containing Plant Extract on Streptozotocin-Treated and Goto-Kakizaki Rats	Fujii et al. (2009)	Japan
	[47] Antidiabetic Effect of Total Flavonoids from *Sanguis d raxonis* in Type 2 Diabetic Rats	Chen et al. (2013)	China
	[48] Antidiabetic Effects of Bitter Gourd Extracts in Insulin-Resistant db/db Mice	Klomann et al. (2010)	Germany
	[49] Anti-Diabetic Effects of *Centratherum anthelminticum* Seeds Methanolic Fraction on Pancreatic Cells, Β-TC6 and Its Alleviating Role in Type 2 Diabetic Rats	Arya et al. (2012)	Malaysia
	[17] Anti-Diabetic Effects of Polysaccharides from *Talinum triangulare* in Streptozotocin (STZ)-Induced Type 2 Diabetic Male Mice	Xu et al. (2015)	China
	[50] Anti-Diabetic Effects of the Acetone Fraction of *Senna singueana* Stem Bark in a Type 2 Diabetes Rat Model	Ibrahim and Islam (2014)	South Africa
	[51] Antidiabetic Potential of Polysaccharides from the White Oyster Culinary-Medicinal Mushroom *Pleurotus florida* (Higher Basidiomycetes)	Ganeshpurkar et al. (2014)	India
	[52] Antihyperglycemic Effects of Total Flavonoids from *Polygonatum odoratum* in STZ and Alloxan-Induced Diabetic Rats	Shu et al. (2009)	China
	[53] Antioxidant and Anti-Inflammatory Effects of a Hypoglycemic Fraction from *Cucurbita ficifolia* Bouché in Streptozotocin-Induced Diabetic Mice	Roman-Ramos et al. (2012)	Mexico
	[54] Effect of *Lycium barbarum *Polysaccharide on the Improvement of Insulin Resistance in NIDDM Rats	Zhao et al. (2005)	China
	[55] Effects of Grape Seed Extract and Its Ethylacetate/Ethanol Fraction on Blood Glucose Levels in a Model of Type 2 Diabetes	Hwang et al. (2009)	South Korea
	[56] Hypoglycemic Effect of *Astragaluspolysaccharide* and Its Effect on PTP1B1	Wu et al. (2005)	China
	[57] Hypoglycemic Effects of MDG-1, a Polysaccharide Derived from *Ophiopogon japonicus*, in the ob/ob Mouse Model of Type 2 Diabetes Mellitus	Xu et al. (2011)	China
	[58] Isolation and Pharmacological Activities of the *Tecoma stans* Alkaloids	Costantino et al. (2003)	Italy
	[58] Potent Effects of the Total Saponins From *Dioscorea nipponica* Makino against Streptozotocin-Induced Type 2 Diabetes Mellitus in Rats	Yu et al. (2015)	China
	[59] Tocopherol from Seeds of *Cucurbita pepo* against Diabetes: Validation by In Vivo Experiments Supported by Computational Docking	Bharti et al. (2013)	India
	[60] *Trapa natans* L. Root Extract Suppresses Hyperglycemic and Hepatotoxic Effects in STZ-Induced Diabetic Rat Model	Kharbanda et al. (2014)	India

**(b):** 

	Title	Animal model/strain	Number of animals	Sex	Age	Weight	Housing of animals	Animals per cage	Groups and number of animals in each group	Randomization
Isolates	[25] A Polysaccharide Extract of Mulberry Leaf Ameliorates Hepatic Glucose Metabolism and Insulin Signaling in Rats with Type 2 Diabetes Induced by High Fat-Diet and Streptozotocin	Wistar rats	18	M	7 w	200 g	Cages	5	3 groups (6 in each group)	Yes
	[26] Antidiabetic Activity of a Xanthone Compound, Mangiferin	Mice	?	M	12 w	?	?	?	?	?
	[27] Anti-Diabetic Effect of a Novel N-Trisaccharide Isolated from *Cucumis prophetarum *on Streptozotocin-Nicotinamide Induced Type 2 Diabetic Rats	Wistar rats	36	M&F	?	150–180 g	Cages	?	6 groups (6 animals in each)	?
	[28] Antidiabetic Effects of the *Cimicifuga racemosa* Extract Ze 450 *In Vitro* and *In Vivo* in ob/ob Mice	ob/ob mice	68	M	7-8 w	?	Cages	1	8 groups (8 animals in each)	?
	[29] Borapetoside C from *Tinospora crispa* Improves Insulin Sensitivity in Diabetic Mice	Mice	?	M	8–10 w	?	?	?	?	?
	[30] Dehydrotrametenolic Acid Induces Preadipocyte Differentiation and Sensitizes Animal Models of Noninsulin-Dependent Diabetes Mellitus to Insulin	C57BLK mice	?	M	7 w	?	?	?	?	?
	[31] Effects of Ingested Fruiting Bodies, Submerged Culture Biomass, and Acidic Polysaccharide Glucuronoxylomannan of *Tremella mesenterica* Retz.:Fr. on Glycemic Responses in Normal and Diabetic Rats	Wistar rats	102	M	?	200 g	Cages	1	6 groups (12 animals in each group) and 3 groups (10 animals in each group)	Yes
	[32] Inhibition of Glycogen Synthase Kinase-3*β* by Falcarindiol Isolated from Japanese Parsley (*Oenanthe javanica*)	GK rats	12	M	?	?	Cages	1	2 groups (6 animals in each group)	?
	[33] Isolation and Antihyperglycemic Activity of Bakuchiol from *Otholobium pubescens* (Fabaceae), a Peruvian Medicinal Plant Used for the Treatment of Diabetes	C57BL/K mice & Sprague- Dawley rats	?	M	8 w	?	?	4	? Groups (8 animals in each group)	?
	[34] LBP-4a Improves Insulin Resistance via Translocation and Activation of GLUT4 in OLETF Rats	Otsuka Long-Evans Tokushima Fatty (OLETF) rats	18	M	26 w	?	?	?	3 groups (6 animals in each group)	Yes
	[35] Meliacinolin: A Potent Α-Glucosidase and Α-Amylase Inhibitor Isolated from *Azadirachta indica* Leaves and In Vivo Antidiabetic Property in Streptozotocin-Nicotinamide-Induced Type 2 Diabetes in Mice	?/mice	42	M	?	20–25 g	Cages	2	7 groups (6 animals in each group)	?
	[36] Novel Terpenoid-Type Quinones Isolated from *Pycnanthus angolensis* of Potential Utility in the Treatment of Type 2 Diabetes	C57BLK mice	?	M	7-8 w	?	Cages	4	? groups (5–8 animals in each group)	?
	[37] Platyconic Acid, a Saponin from *Platycodi radix*, Improves Glucose Homeostasis by Enhancing Insulin Sensitivity In Vitro and In Vivo	C57BLK mice	90	M	8–10 w	?	?	1	6 groups (15 animals in each group)	Yes
	[38] Rhaponticin from rhubarb Rhizomes Alleviates Liver Steatosis and Improves Blood Glucose and Lipid Profiles in KK/Ay Diabetic Mice	Mice	30	M&F	8–12 w	?	Cages	1	3 groups (10 animals in each group)	Yes
	[39] Rutin Potentiates Insulin Receptor Kinase to Enhance Insulin-Dependent Glucose Transporter 4 Translocation	Mice	?	M	8 w	?	?	?	?	?
	[40] Type 2 Antidiabetic Activity of Bergenin from the Roots of *Caesalpinia digyna* Rottler	Charles Foster Albino rats	30	M	?	?	?	?	5 groups (6 animals in each group)	?

Fractions	[41] A Study on Hypoglycaemic Health Care Function of *Stigma maydis* Polysaccharides	SPF km mice	?	M	?	20 ± 2 g	?	?	7 groups (animals in each group?)	Yes
	[1] Antidiabetic Activities of Extract from *Malva verticillata *Seed via the Activation of AMP-Activated Protein Kinase.	C57BLK mice	?	?	5 w	?	Cages	5	5 groups (animals in each group?)	?
	[42] Antidiabetic Activity of Alkaloids of *Aerva lanata* Roots on Streptozotocin-Nicotinamide Induced Type-II Diabetes in Rats	Wistar rats	?	M	?	200–250 g	?	?	?	?
	[43] Antidiabetic Activity of *Pterospermum acerifolium *Flowers and Glucose Uptake Potential of Bioactive Fraction in L6 Muscle Cell Lines with Its HPLC Fingerprint	Albino Wistar rats	66	M	?	180–200 g	Cages	?	11 groups (6 animals in each group)	?
	[44] Antidiabetic Activity of *Caesalpinia bonducella* F. in Chronic Type 2 Diabetic Model in Long-Evans Rats and Evaluation of Insulin Secretagogue Property of Its Fractions on Isolated Islets	Long-Evans rats	?	M&F	12–14 w	150 g	?	?	5 groups (6–9 animals in each group)	?
	[45] Antidiabetic Effect of an Acidic Polysaccharide (TAP) from *Tremella aurantia *and Its Degradation Product (TAP-H)	KK- Ay-TA mice	?	M	5 w	25–30 g	?	1	3 groups (animals in each group?)	?
	[46] Antidiabetic Effect of Orally Administered Conophylline-Containing Plant Extract on Streptozotocin-Treated and Goto-Kakizaki Rats	Goto-Kakizaki rats	14	M	5 w	?	?	?	3 groups (animals in each group?)	Yes
	[47] Antidiabetic Effect of Total Flavonoids from *Sanguis draxonis* in Type 2 Diabetic Rats	Sprague-Dawley rats	72	M	?	180–220 g	?	?	6 groups (6 animals in each group) for SD; 6 groups (6 animals in each group) for SDF	?
	[48] Antidiabetic Effects of Bitter Gourd Extracts in Insulin-Resistant db/db Mice	db/db mice	45	M	5 w	?	Cages	1	5 groups (9 animals in each group)	?
	[49] Anti-Diabetic Effects of *Centratherum anthelminticum* Seeds Methanolic Fraction on Pancreatic Cells, Β-TC6 and Its Alleviating Role in Type 2 Diabetic Rats	Sprague-Dawley rats	72	M&F	?	180–200 g	?	?	12 groups (6 animals in each group)	?
	[17] Anti-Diabetic Effects of Polysaccharides from *Talinum triangulare* in Streptozotocin (STZ)-Induced Type 2 Diabetic Male Mice	SPF km mice	50	M	?	20 ± 2 g	?	?	5 groups (10 animals in each group)	Yes
	[50] Anti-Diabetic Effects of the Acetone Fraction of *Senna singueana* Stem Bark in a Type 2 Diabetes Rat Model	Sprague-Dawley rats	48	M	6 w	207.60 ± 4.27 g	Cage	2	6 groups (8 animals in each group)	Yes
	[51] Antidiabetic Potential of Polysaccharides from the White Oyster Culinary-Medicinal Mushroom *Pleurotus florida* (Higher Basidiomycetes)	Wistar rats	20	M	?	150–200 g	?	?	4 groups (5 animals in each group)	?
	[52] Antihyperglycemic Effects of Total Flavonoids from *Polygonatum odoratum* in STZ and Alloxan-Induced Diabetic Rats	Sprague-Dawley rats	?	?	?	220 ± 4.5 g	Special animal house	?	?	?
	[53] Antioxidant and Anti-Inflammatory Effects of a Hypoglycemic Fraction from *Cucurbita ficifolia* Bouché in Streptozotocin-Induced Diabetic Mice	CD-1 mice	?	M	?	30–35 g	?	?	?	?
	[54] Effect of *Lycium barbarum *Polysaccharide on the Improvement of Insulin Resistance in NIDDM Rats	Wistar rats	40	M	?	230–250 g	Cages	?	?	Yes
	[55] Effects of Grape Seed Extract and Its Ethylacetate/Ethanol Fraction on Blood Glucose Levels in a Model of Type 2 Diabetes	C57BL/KsJ-leprdb/leprdb mice	98	M	3 w	9.7–14.2 g	Conventional state	?	7 groups (14 in each group)	?
	[56] Hypoglycemic Effect of *Astragaluspolysaccharide* and Its Effect on PTP1B1	Sprague-Dawley rat	34	M	8 w	200 g	?	5	4 groups (2 groups with 10 animals and 2 groups with 12 animals in each)	?
	[57] Hypoglycemic Effects of MDG-1, a Polysaccharide Derived from *Ophiopogon j aponicus*, in the ob/ob Mouse Model of Type 2 Diabetes Mellitus	ob/ob mice	?		6-7 w	?	?	?	4 groups (8 animals in each group)	?
	[58] Isolation and Pharmacological Activities of the *Tecoma stans* Alkaloids	C57BL/KsJ db/db mice	?	M	8 w	?	?	?	?	?
	[61] Potent Effects of the Total Saponins from *Dioscorea nipponica* Makino against Streptozotocin-Induced Type 2 Diabetes Mellitus in Rats	Wistar rats	70	M	?	190–200 g	Cages	1	7 groups (8 animals in each group)	Yes
	[59] Tocopherol from Seeds of *Cucurbita pepo* against Diabetes: Validation by In Vivo Experiments Supported by Computational Docking	Albino Wistar rats	24	M	?	150–160 g	?	?	4 groups (6 animals in each group)	?
	[60] *Trapa natans* L. Root Extract Suppresses Hyperglycemic and Hepatotoxic Effects in STZ-Induced Diabetic Rat Model	Albino Wistar rats	90	?	?	150–200 g	Cages	?	15 groups (6 animals in each group)	?

**(c):** 

	Title	Plant species	Isolate/fraction	Administration	Doses used	Duration of treatment
Isolates	[25] A Polysaccharide Extract of Mulberry Leaf Ameliorates Hepatic Glucose Metabolism and Insulin Signaling in Rats with Type 2 Diabetes Induced by High Fat-Diet and Streptozotocin	Mulberry	Mulberry leaf polysaccharide	Gavage	200 mg/kg	6 days of treatment
	[26] Antidiabetic Activity of a Xanthone Compound, Mangiferin	*Anemarrhena asphodeloides *Bunge	Mangiferin	Orally	30 mg/kg	?
	[27] Anti-Diabetic Effect of a Novel N-Trisaccharide Isolated from *Cucumis prophetarum* on Streptozotocin-Nicotinamide Induced Type 2 Diabetic Rats	*Cucumis prophetarum*	N-Trisaccharide	Gavage	50, 5 mg/kg	28 days of treatment
	[28] Antidiabetic Effects of the *Cimicifuga racemosa* Extract Ze 450 *In Vitro* and *In Vivo* in ob/ob Mice	*Cimicifuga racemosa*	Ze 450	Gavage	10, 30, 90 mg/kg	7 days of treatment
	[29] Borapetoside C from *Tinospora crispa* Improves Insulin Sensitivity in Diabetic Mice	*Tinospora crispa *	Borapetoside C	Orally	5 mg/kg	4 weeks of treatment
	[30] Dehydrotrametenolic Acid Induces Preadipocyte Differentiation and Sensitizes Animal Models of Noninsulin-Dependent Diabetes Mellitus to Insulin	*Poria cocos *Wolf	Dehydrotrametenolic acid	Gavage	110 mg/kg	14 days of treatment
	[31] Effects of Ingested Fruiting Bodies, Submerged Culture Biomass, and Acidic Polysaccharide Glucuronoxylomannan of *Tremella mesenterica* Retz.:Fr. on Glycemic Responses in Normal and Diabetic Rats	*Tremella mesenterica*	Acidic polysaccharide glucuronoxylomannan (GXM)	Gavage	1 g/kg	15 days of treatment
	[32] Inhibition of Glycogen Synthase Kinase-3*β* by Falcarindiol Isolated from Japanese Parsley (*Oenanthe javanica*)	*Oenanthe javanica*	falcarindiol	Orally	15 mg/kg	?
	[33] Isolation and Antihyperglycemic Activity of Bakuchiol from *Otholobium pubescens* (Fabaceae), a Peruvian Medicinal Plant Used for the Treatment of Diabetes	*Otholobium pubescens*	Bakuchiol	Gavage	1, 150, 250 mg/kg	2 weeks of treatment
	[34] LBP-4a Improves Insulin Resistance via Translocation and Activation of GLUT4 in OLETF Rats	*Lycium barbarum*	*Lycium barbarum* polysaccharide (LBP-4a)	Orally	10 mg/kg	4 weeks of treatment
	[35] Meliacinolin: A Potent Α-Glucosidase and Α-Amylase Inhibitor Isolated from *Azadirachta indica* Leaves and In Vivo Antidiabetic Property in Streptozotocin-Nicotinamide-Induced Type 2 Diabetes in Mice	*Azadirachta indica*	Meliacinolin	Orally by gastric intubations	20 mg/kg	28 days of treatment
	[36] Novel Terpenoid-Type Quinones Isolated from *Pycnanthus angolensis* of Potential Utility in the Treatment of Type 2 Diabetes	*Pycnanthus angolensis *	Novel terpenoid-type quinones (SP-18904 and SP-18905)	Gavage	100 mg/kg	4 days of treatment
	[37] Platyconic Acid, a Saponin from *Platycodi radix*, Improves Glucose Homeostasis by Enhancing Insulin Sensitivity In Vitro and In Vivo	*Platycodi radix*	Platyconic acid (PA), platycodin D (PD), platycoside E (PE), and saponin with low activity (DPE)	Gavage	20 mg/kg	8 weeks of treatment
	[38] Rhaponticin from rhubarb Rhizomes Alleviates Liver Steatosis and Improves Blood Glucose and Lipid Profiles in KK/Ay Diabetic Mice	*Rheum franzenbachii *Munt	Rhaponticin	Orally	125 mg/kg	4 weeks of treatment
	[39] Rutin Potentiates Insulin Receptor Kinase to Enhance Insulin-Dependent Glucose Transporter 4 Translocation	*Toona sinensis*	Flavonoid rutin	Gavage	25 mg/kg	?
	[40] Type 2 Antidiabetic Activity of Bergenin from the Roots of *Caesalpinia digyna* Rottler	*Caesalpinia digyna *Rottler	Bergenin	Orally	2.5, 5, and 10 mg/kg	14 days of treatment

Fractions	[41] A Study on Hypoglycaemic Health Care Function of *Stigma maydis* Polysaccharides	*Stigma maydis*	*Stigma maydis* polysaccharides	?	20 mg/kg	4 weeks of treatment
	[1] Antidiabetic Activities of Extract from *Malva verticillata* Seed via the Activation of AMP-Activated Protein Kinase	*Malva verticillata *	Ethanol extract of *M. verticillata* and N-hexane (MVE-H)	Orally	3 different concentrations of MVE-H (10, 20, or 40 mg/kg)	4 weeks of treatment
	[42] Antidiabetic Activity of Alkaloids of *Aerva lanata* Roots on Streptozotocin-Nicotinamide Induced Type-II Diabetes in Rats	*Aerva lanata *Linn.	The partially purified alkaloid basified toluene fraction (PPABTF)	Orally	10, 20 mg/kg	2 weeks of treatment
	[43] Antidiabetic Activity of *Pterospermum acerifolium* Flowers and Glucose Uptake Potential of Bioactive Fraction in L6 Muscle Cell Lines with Its HPLC Fingerprint	*Pterospermum acerifolium*	Ethyl acetate fraction (PAFEF) and subfractions PAFE1, PAFE2, and e PAFE3	Intragastric tube	200, 400 mg/kg, and 15, 30 mg/kg	30 days of treatment
	[44] Antidiabetic Activity of *Caesalpinia bonducella* F. in Chronic Type 2 Diabetic Model in Long-Evans Rats and Evaluation of Insulin Secretagogue Property of Its Fractions on Isolated Islets	*Caesalpinia bonducella *F.	*Caesalpinia bonducella *aqueous and alcoholic extracts (BM-170 and BM-171)	Orally	250 mg/kg	28 days of treatment
	[45] Antidiabetic Effect of an Acidic Polysaccharide (TAP) from *Tremella aurantia *and Its Degradation Product (TAP-H)	*Tremella aurantia*	Acidic polysaccharide (TAP) and the degradation product (TAP-H)	Orally	0.5 g/L, 1.5 g/L	10 weeks of treatment
	[46] Antidiabetic Effect of Orally Administered Conophylline-Containing Plant Extract on Streptozotocin-Treated and Goto-Kakizaki Rats	*Tabernaemontana divaricata*	The Crude Conophylline Preparation I (CCP-I)	Orally	200, 50 g/kg	15 days of treatment
	[47] Antidiabetic Effect of Total Flavonoids from *Sanguis draxonis* in Type 2 Diabetic Rats	*Dracaena cochinchinensis*	*Sanguis draxonis* (SD) and total flavonoids from SD (SDF)	Gavage	?	21 days of treatment
	[48] Antidiabetic Effects of Bitter Gourd Extracts in Insulin-Resistant db/db Mice	*Momordica charantia*	The lipid fraction, the saponin fraction, or the hydrophilic residue of bitter gourd	Orally	150 mg/kg	5 weeks of treatment
	[49] Anti-Diabetic Effects of *Centratherum anthelminticum* Seeds Methanolic Fraction on Pancreatic Cells, Β-TC6 and Its Alleviating Role in Type 2 Diabetic Rats	*Centratherum anthelminticum*	Crude methanolic fraction (CAMF)	Injected intraperitoneally (IP) or orally	50 and 100 mg/kg	4 weeks of treatment
	[17] Anti-Diabetic Effects of Polysaccharides from *Talinum triangulare* in Streptozotocin (STZ)-Induced Type 2 Diabetic Male Mice	*Talinum triangulare*	Polysaccharides obtained from *Talinum triangulare* (TTP)	Orally	150 and 300 mg/kg	2 weeks of treatment
	[50] Anti-Diabetic Effects of the Acetone Fraction of *Senna singueana* Stem Bark in a Type 2 Diabetes Rat Model	*Senna singueana *	*Senna singueana* acetone fraction (SSAF)	Orally	150 mg/kg and 300 mg/kg	4 weeks of treatment
	[51] Antidiabetic Potential of Polysaccharides from the White Oyster Culinary-Medicinal Mushroom *Pleurotus florida* (Higher Basidiomycetes)	*Pleurotus florida*	*P. florida* polysaccharides (PFPs)	Orally	200 and 400 mg/kg	21 days of treatment
	[52] Antihyperglycemic Effects of Total Flavonoids from *Polygonatum odoratum* in STZ and Alloxan-Induced Diabetic Rats	*Polygonatum odoratum*	Total flavonoids of *Polygonatum* (P) *odoratum* (TFP)	Orally	50, 100, and 200 mg/kg	30 days of treatment
	[53] Antioxidant and Anti-Inflammatory Effects of a Hypoglycemic Fraction from *Cucurbita ficifolia* Bouché in Streptozotocin-Induced Diabetic Mice	*Cucurbita ficifolia*	Aqueous-precipitate fraction (AP-fraction)	Gavage	200 mg/kg	15 days of treatment
	[54] Effect of *Lycium barbarum *Polysaccharide on the Improvement of Insulin Resistance in NIDDM Rats	*Lycium barbarum*	*Lycium barbarum* polysaccharide (LBP)	Orally	10 mg/kg	3 weeks of treatment
	[55] Effects of Grape Seed Extract and Its Ethylacetate/Ethanol Fraction on Blood Glucose Levels in a Model of Type 2 Diabetes	*Vitis vinifera *grape	Grape seed extract (GSE), ethylacetate (e), and ethylacetate/ethanol (ee)	Orally	50 and 30 mg/kg	8 weeks of treatment
	[56] Hypoglycemic Effect of *Astragaluspolysaccharide* and Its Effect on PTP1B1	*Astragalus membranaceus*	*Astragalus polysaccharide* (APS)	Orally	400 mg/kg	5 weeks of treatment
	[57] Hypoglycemic Effects of MDG-1, a Polysaccharide Derived from *Ophiopogon japonicus*, in the ob/ob Mouse Model of Type 2 Diabetes Mellitus	*Ophiopogon japonicus*	Water-soluble *β*-d-fructan (MDG-1)	Intragastrically	150 and 300 mg/kg	23 days of treatment
	[58] Isolation and Pharmacological Activities of the *Tecoma stans* Alkaloids	*Tecoma stans *	*Tecoma stans* alkaloids	Gavage	50 mg/kg and 63.4 mg/kg	7 days of treatment
	[61] Potent Effects of the Total Saponins from *Dioscorea nipponica* Makino against Streptozotocin-Induced Type 2 Diabetes Mellitus in Rats	*Dioscorea nipponica*	Saponins from *D. nipponica *Makino (TSDN)	Orally	200, 100, and 50 mg/kg	12 weeks of treatment
	[59] Tocopherol from Seeds of *Cucurbita pepo* against Diabetes: Validation by In Vivo Experiments Supported by Computational Docking	* Cucurbita pepo*	Tocopherol	?	2, 5 g/kg	6 weeks of treatment
	[60] *Trapa natans* L. Root Extract Suppresses Hyperglycemic and Hepatotoxic Effects in STZ-Induced Diabetic Rat Model	*Trapa natans *L.	Methanol fraction, chloroform fraction, and petroleum ether fraction	Orally	50, 100, and 200 mg/kg	15 days of treatment

**(d):** 

	Title	Drug for diabetes induction	Route of induction	Hyperglycemia	Control of glycemia	Insulin tolerance test
Isolates	[25] A Polysaccharide Extract of Mulberry Leaf Ameliorates Hepatic Glucose Metabolism and Insulin Signaling in Rats with Type 2 Diabetes Induced by High Fat-Diet and Streptozotocin	Diet with 41.2% fat and a low-dose STZ (35 mg/kg body weight)	Intraperitoneal	Glucose ≥ 7.8 mmol/L	?	Yes
	[26] Antidiabetic Activity of a Xanthone Compound, Mangiferin	Genetically modified	—	—	?	Yes
	[27] Anti-Diabetic Effect of a Novel N-Trisaccharide Isolated from *Cucumis prophetarum* on Streptozotocin-Nicotinamide Induced Type 2 Diabetic Rats	Nicotinamide (NA) at 230 mg/kg and STZ at 65 mg/kg	Intraperitoneal	Glucose ≥ 250 mg/dL	Yes	Yes
	[28] Antidiabetic Effects of the *Cimicifuga racemosa* Extract Ze 450 *In Vitro* and *In Vivo* in ob/ob Mice	Genetically modified	—	—	?	?
	[29] Borapetoside C from *Tinospora crispa* Improves Insulin Sensitivity in Diabetic Mice	STZ 150 mg/kg	Intraperitoneal	Glucose ≥ 150 mg/dL	Yes	Yes
	[30] Dehydrotrametenolic Acid Induces Preadipocyte Differentiation and Sensitizes Animal Models of Noninsulin-Dependent Diabetes Mellitus to Insulin	Genetically modified	—	—	?	?
	[31] Effects of Ingested Fruiting Bodies, Submerged Culture Biomass, and Acidic Polysaccharide Glucuronoxylomannan of *Tremella mesenterica* Retz.:Fr. on Glycemic Responses in Normal and Diabetic Rats	STZ (65 mg/kg) and nicotinamide (200 mg/kg)	Intraperitoneal	Glucose > 250 mg/100 mL	Yes	Yes
	[32] Inhibition of Glycogen Synthase Kinase-3*β* by Falcarindiol Isolated from Japanese Parsley (*Oenanthe javanica*)	Genetically modified	—	—	?	Yes
	[33] Isolation and Antihyperglycemic Activity of Bakuchiol from *Otholobium pubescens* (Fabaceae), a Peruvian Medicinal Plant Used for the Treatment of Diabetes	STZ 50 mg/kg	Intravenous	Glucose 300–600 mg/dL	Yes	?
	[34] LBP-4a Improves Insulin Resistance via Translocation and Activation of GLUT4 in OLETF Rats	Genetically modified	—	—	?	Yes
	[35] Meliacinolin: A Potent Α-Glucosidase and Α-Amylase Inhibitor Isolated from *Azadirachta indica* Leaves and In Vivo Antidiabetic Property in Streptozotocin-Nicotinamide-Induced Type 2 Diabetes in Mice	Nicotinamide (120 mg/kg) and STZ (60 mg/kg)	Intraperitoneal	Glucose > 250 mg/dL	Yes	?
	[36] Novel Terpenoid-Type Quinones Isolated from *Pycnanthus angolensis* of Potential Utility in the Treatment of Type 2 Diabetes	STZ 150 mg/kg	Intravenous	Glucose 300–600 mg/dL	Yes	Yes
	[37] Platyconic Acid, a Saponin from *Platycodi radix*, Improves Glucose Homeostasis by Enhancing Insulin Sensitivity *In Vitro* and *In Vivo*	STZ 20 mg/kg	?	?	Yes	Yes
	[38] Rhaponticin from rhubarb Rhizomes Alleviates Liver Steatosis and Improves Blood Glucose and Lipid Profiles in KK/Ay Diabetic Mice	STZ/?	?	?	Yes	?
	[39] Rutin Potentiates Insulin Receptor Kinase to Enhance Insulin-Dependent Glucose Transporter 4 Translocation	Insulin receptor antagonist S960 (50 nmol/kg)	Intravenous injection	?	Yes	?
	[40] Type 2 Antidiabetic Activity of Bergenin from the Roots of *Caesalpinia digyna* Rottler	STZ (65 mg/kg) and nicotinamide (110 mg/kg)	Intraperitoneal injection	Glucose 200 mg/dL	Yes	Yes

Fractions	[41] A Study on Hypoglycaemic Health Care Function of *Stigma maydis* Polysaccharides	High fat-diet and low-dose alloxan (90 mg/kg)	Intraperitoneal	Glucose ≥ 10 mmol/L	Yes	?
	[1] Antidiabetic Activities of Extract from *Malva verticillata* Seed via the Activation of AMP-Activated Protein Kinase	Genetically modified	—	—	Yes	?
	[42] Antidiabetic Activity of Alkaloids of *Aerva lanata* Roots on Streptozotocin-Nicotinamide Induced Type-II Diabetes in Rats	STZ and nicotinamide	Intraperitoneal	?	Yes	?
	[43] Antidiabetic Activity of *Pterospermum acerifolium* Flowers and Glucose Uptake Potential of Bioactive Fraction in L6 Muscle Cell Lines with Its HPLC Fingerprint	STZ (60 mg/kg) and nicotinamide (120 mg/kg)	Intraperitoneal	Glucose > 250 mg/dL	Yes	?
	[44] Antidiabetic Activity of *Caesalpinia bonducella* F. in Chronic Type 2 Diabetic Model in Long-Evans Rats and Evaluation of Insulin Secretagogue Property of Its Fractions on Isolated Islets	STZ 90 mg/kg	Intraperitoneal injection	Glucose 7–12 mmol/L	Yes	Yes
	[45] Antidiabetic Effect of an Acidic Polysaccharide (TAP) from *Tremella aurantia *and Its Degradation Product (TAP-H)	Genetically modified	—	—	?	Yes
	[46] Antidiabetic Effect of Orally Administered Conophylline-Containing Plant Extract on Streptozotocin-Treated and Goto-Kakizaki Rats	Streptozotocin 60 mg/kg	Intraperitoneal	Glucose > 250 mg/dL	Yes	?
	[47] Antidiabetic Effect of Total Flavonoids from *Sanguis draxonis* in Type 2 Diabetic Rats	High fat-diet and a singular injection of streptozotocin (STZ) (35 mg/kg)	Intraperitoneal	Glucose ≥ 11.1 mmol/L	Yes	Yes
	[48] Antidiabetic Effects of Bitter Gourd Extracts in Insulin-Resistant db/db Mice	Genetically modified	—	—	?	?
	[49] Anti-Diabetic Effects of *Centratherum anthelminticum* Seeds Methanolic Fraction on Pancreatic Cells, Β-TC6 and Its Alleviating Role in Type 2 Diabetic Rats	Streptozotocin (STZ) (65 mg/kg)	Intraperitoneal	Glucose 22–26 mmol/L	Yes	?
	[17] Anti-Diabetic Effects of Polysaccharides from *Talinum triangulare* in Streptozotocin (STZ)-Induced Type 2 Diabetic Male Mice	Streptozotocin (STZ) (70 mg/kg)	Intraperitoneal	?	Yes	?
	[50] Anti-Diabetic Effects of the Acetone Fraction of *Senna singueana* Stem Bark in a Type 2 Diabetes Rat Model	STZ 40 mg/kg	Intraperitoneal injection	Glucose > 18 mmol/L	Yes	?
	[51] Antidiabetic Potential of Polysaccharides from the White Oyster Culinary-Medicinal Mushroom *Pleurotus florida* (Higher Basidiomycetes)	STZ 50 mg/kg	Intraperitoneal	Glucose > 200 mg/100 mL	Yes	?
	[52] Antihyperglycemic Effects of Total Flavonoids from *Polygonatum odoratum* in STZ and Alloxan-Induced Diabetic Rats	Alloxan (100 and 120 mg/kg)	Intraperitoneal	Glucose > 11.0 mmol/L	Yes	?
	[53] Antioxidant and Anti-Inflammatory Effects of a Hypoglycemic Fraction from *Cucurbita ficifolia*Bouché in Streptozotocin-Induced Diabetic Mice	STZ 137 mg/kg	Intraperitoneal	?	?	?
	[54] Effect of *Lycium barbarum *Polysaccharide on the Improvement of Insulin Resistance in NIDDM Rats	STZ 50 mg/kg	Intraperitoneal	Glucose > 16 mmol/L	Yes	Yes
	[55] Effects of Grape Seed Extract and Its Ethylacetate/Ethanol Fraction on Blood Glucose Levels in a Model of Type 2 Diabetes	Genetically modified	—	—	Yes	?
	[56] Hypoglycemic Effect of *Astragalus* Polysaccharide and Its Effect on PTP1B1	STZ 30 mg/kg	Intravenous injection	Glucose > 6.7 mmol/L	Yes	Yes
	[57] Hypoglycemic Effects of MDG-1, a Polysaccharide Derived from *Ophiopogon japonicus*, in the ob/ob Mouse Model of Type 2 Diabetes Mellitus	Genetically diabetic model	—	—	Yes	Yes
	[58] Isolation and Pharmacological Activities of the *Tecoma stans* Alkaloids	Genetically diabetic model	—	—	?	Yes
	[61] Potent Effects of the Total Saponins from *Dioscorea nipponica* Makino against Streptozotocin-Induced Type 2 Diabetes Mellitus in Rats	STZ 30 mg/kg	Intraperitoneal	Glucose > 16.7 mmol/L	Yes	Yes
	[59] Tocopherol from Seeds of *Cucurbita pepo* against Diabetes: Validation by In Vivo Experiments Supported by Computational Docking	PX-407 solution (10 mg/kg)	?	Blood glucose level of 200 mg/dL or higher	Yes	Yes
	[60] *Trapa natans* L. Root Extract Suppresses Hyperglycemic and Hepatotoxic Effects in STZ-Induced Diabetic Rat Model	STZ (45 mg/kg)	Intraperitoneal	Glucose > 200 mg/dL	Yes	?

**Table 2:** Biases analyses (ARRIVE) of the studies of the effects of fractions and isolates from the plants in the treatment of the type II diabetes.

	Isolates																	Fractions																							
References	Miura, et al., 2001 [26]	Kavishankar and Lakshmidevi, 2014 [27]	Moser et al., 2014 [28]	Chen et al., 2009 [38]	Ruan et al., 2012 [29]	Sato et al., 2002 [30]	Lo et al., 2006 [31]	Yoshida et al., 2013 [32]	Zhao et al., 2014 [34]	Kumar et al., 2012 [40]	Luo et al., 1999 [36]	Perez-Gutierrez and Damian-Guzman, 2012 [35]	Kwon et al., 2012 [37]	Krenisky et al., 1999 [33]	Hsu et al., 2014 [39]	Ren et al., 2015 [25]	Agrawal et al., 2013 [42]	Zhang et al., 2013 [41]	Jeong and Song, 2011 [1]	Chakrabarti et al., 2005 [44]	Paramaguru et al., 2014 [43]	Kiho et al., 2001 [45]	Fujii et al., 2009 [46]	Chen et al., 2013 [47]	Arya et al., 2012 [49]	Klomann et al., 2010 [48]	Arya et al., 2012 [49]	Xu et al., 2015 [17]	Ibrahim and Islam, 2014 [50]	Ganeshpurkar et al., 2014 [51]	Shu et al., 2009 [52]	Zhao et al., 2005 [54]	Hwang et al., 2009 [55]	Wu et al., 2005 [56]	Xu et al., 2011 [57]	Costantino et al., 2003 [58]	Yu et al., 2015 [61]	Kharbanda et al., 2014 [60]	Roman-Ramos et al., 2012 [53]		
Accurate and concise description of the content of the article		X	X	X	X	X	X	X	X	X	X		X	X		X	X			X	X		X	X	X	X	X	X	X	X	X	X	X	X	X	X	X	X	X	33	84.62%

*Abstract*																																									
Summary of the background, research objectives, methods, main findings, and conclusions	X	X	X	X	X		X	X	X	X	X	X	X	X	X	X	X	X	X			X	X	X	X	X	X	X	X	X	X	X	X	X	X	X	X	X	X	36	92.30%

*Introduction*																																									
Sufficient scientific background	X	X	X	X	X		X	X	X	X		X	X	X	X	X	X	X	X	X	X	X	X	X	X	X	X	X	X	X	X	X	X	X	X	X	X	X	X	37	94.87%
Explanation of the experimental approach and rationale		X	X	X	X	X	X	X	X	X	X	X	X	X	X	X	X	X	X	X	X	X	X	X	X	X	X	X	X	X	X	X	X	X	X	X	X		X	37	94.87%
*Objectives*																																									
Clear primary and second objectives	X	X	X	X			X	X	X	X	X		X	X	X	X	X	X		X	X	X	X			X	X	X	X	X	X	X	X	X		X	X	X	X	32	82.05%

*Material and methods*																																									
*Ethical statement*																																									
Nature of the ethical review permissions and institutional guidelines for the care and use of animals		X	X	X	X	X	X	X	X	X	X	X	X	X	X	X	X	X	X		X	X		X	X	X	X	X	X	X	X	X	X	X	X	X	X	X	X	36	92.3%
*Study design*																																									
Number of animals per group		X	X	X			X	X	X	X	X	X	X	X		X				X	X			X	X	X	X	X	X	X		X	X	X	X	X	X	X	X	29	74.35%
Information on whether the experiment was performed as a blind controlled study					X			X															X				X						X				X			6	15.38%
*Experimental procedures*																																									
Treatment	X	X	X	X	X	X	X	X	X	X	X	X	X	X	X	X	X	X	X	X	X	X	X	X	X	X	X	X	X	X	X	X	X	X	X	X	X	X	X	39	100.00%
Dosage of treatment	X	X	X	X	X	X	X	X	X	X	X	X		X	X	X	X	X	X	X	X	X	X		X	X	X	X	X	X	X	X	X	X	X	X	X	X	X	37	94.87%
Route of administration	X	X	X	X	X	X	X	X	X	X	X	X	X	X	X	X	X		X	X	X	X	X	X	X	X	X	X	X	X	X	X	X	X	X	X	X	X	X	38	97.44%
Duration of treatment	X	X	X	X		X	X			X	X	X		X	X	X	X	X	X	X	X	X	X	X	X	X	X	X	X	X	X	X	X	X	X	X	X	X	X	35	89.74%
Time of day for treatment administration		X										X						X	X	X			X	X							X					X				9	23.07%
Location used for administration of treatment																												X												1	2.56%
Rationale for choice of specific dosage					X		X		X			X	X		X					X	X		X										X			X			X	12	30.76%
Rationale for choice of specific route of administration					X		X		X																											X		X	X	6	15.38%
*Experimental animals*																																									
Information on animal species	X	X	X	X	X	X	X	X	X	X	X	X	X	X		X	X	X	X	X	X	X	X	X	X	X	X	X	X	X	X	X	X	X			X	X		35	89.74%
Strain of the animals		X			X	X	X	X	X	X	X		X			X	X	X	X	X	X	X	X	X	X	X			X	X	X	X	X	X			X	X	X	29	74.36%
Sex of the animals	X	X	X	X	X	X	X	X	X	X	X	X	X	X	X	X	X	X		X	X	X	X	X	X	X	X	X	X	X		X	X	X		X	X	X	X	36	92.30%
Animals weight range		X					X					X		X		X	X	X		X	X	X		X	X		X	X	X		X	X	X	X			X	X	X	22	56.41%
Age of the animals	X		X	X	X	X			X		X		X	X	X	X			X	X		X	X		X	X			X				X	X	X		X			22	56.41%
Description of genetic modification status (knock-out, transgenic, and SPF)	X		X	X					X	X	X				X			X	X			X	X					X					X	X		X	X	X	X	18	46.15%
Information related to previous procedures performed on the animals		X	X	X	X	X	X	X			X	X	X	X	X			X	X	X	X	X	X	X	X		X							X			X	X	X	25	64.10%
*Housing and husbandry*																																									
Housing of experimental animals (type of facility, type of cage or housing, material, and number of cage companions)			X				X	X			X	X	X			X			X		X					X	X	X	X								X			14	35.89%
Husbandry conditions (breeding programme, light/dark cycle, and temperature of water)		X	X	X	X		X	X		X	X	X	X	X	X	X	X		X	X	X	X	X	X	X	X	X	X	X	X	X	X	X	X	X		X		X	33	84.62%
Welfare-related assessments and interventions that were carried out before, during, or after the experiment							X													X	X		X														X			5	12.82%
*Sample size*																																									
Total number of animals used in each experiment and the number of animals in each experimental group		X	X	X			X	X	X	X		X	X			X					X		X	X	X	X	X	X	X	X		X			X		X			22	56.41%
Explanation regarding the decision of the number of animals and details of sample size calculation							X																										X							2	5.12%
*Allocation of animals into experimental groups*																																									
Full details of how animals were allocated to experimental groups (including randomization or matching)				X			X		X				X					X					X		X			X	X			X					X			11	28.20%
Order in which the animals in the different experimental groups were treated and assessed	X	X	X	X	X		X	X	X		X	X						X	X	X	X	X	X	X	X	X	X	X		X			X	X	X		X	X	X	28	71.79%
*Experimental outcomes*																																									
Clear experimental outcomes assessed		X	X	X	X		X	X	X	X	X	X		X							X	X	X	X	X	X	X	X	X	X	X	X	X	X			X	X	X	28	71.79%
*Statistical methods*																																									
Statistical methods used for each analysis	X	X	X	X	X		X	X	X	X			X	X	X	X	X	X	X	X	X	X	X	X	X	X	X	X	X		X	X	X	X	X		X	X	X	34	87.18%
Specification of the unit of analysis for each dataset				X	X		X	X	X	X		X	X	X	X	X	X	X	X	X	X	X	X	X	X	X	X	X	X	X	X	X	X	X	X		X	X	X	33	84.61%
Methods used to assess whether the data met the assumptions of the statistical approach	X	X	X	X	X		X	X	X	X		X	X	X		X	X	X	X	X	X	X	X	X	X	X	X	X	X	X	X	X	X	X			X	X	X	34	87.18%

*Results*																																									
*Baseline data*																																									
Description of the animals health status, for each experimental group, before treatment	X	X	X	X					X	X								X	X	X	X	X	X	X			X		X			X		X			X			18	46.15%
*Number analyzed*																																									
Number or animals in each group included in each analysis (absolute numbers)							X	X		X						X										X	X		X			X	X	X			X		X	12	30.76%
Animals or data not included in the analysis (and explanation for the exclusion)																																		X						1	2.56%
*Outcomes and estimation*																																									
Information (mean = standard deviation)	X	X	X	X	X							X	X					X	X	X	X	X	X	X	X								X	X			X	X	X	20	51.28%
Information on quantification of inflammatory cells (mean = standard deviation)																								X																1	2.56%
*Adverse events*																																									
Information on mortality of experimental animal (mean = standard deviation)			X																		X											X								3	7.69%
Modifications to the experimental protocols made to reduce adverse events			X																																		X			2	5.12%
*Discussion*																																									
*Interpretation/scientific implications*																																									
Interpretation of the results, taking into account the study objectives and hypotheses	X	X	X	X		X	X	X	X	X	X	X	X	X	X		X	X	X	X	X	X	X	X	X	X	X	X	X	X	X	X	X	X			X	X	X	35	89.74%
Comments on the study limitations (sources of bias and imprecision associated with the results)		X	X			X	X		X	X										X													X	X			X	X	X	12	30.76%
*Generalizability /translation*																																									
Comments on how the findings are likely to translate to other species or systems (relevance to human biology)			X	X	X	X	X	X	X	X		X	X		X	X	X	X					X			X	X						X	X			X	X	X	22	56.41%
*Funding*																																									
List of funding sources and the role of the funder(s) in the study		X	X	X						X			X	X	X	X	X		X	X	X		X	X	X	X	X	X	X	X	X	X	X	X		X	X	X	X	28	71.79%

Results	17	28	31	29	24	15	33	26	28	27	23	25	27	23	20	24	21	24	22	27	29	25	32	27	27	27	29	27	28	22	21	25	32	32	16	17	38	26	30		

## References

[1] Jeong Y.-T., Song C.-H. (2011). Antidiabetic activities of extract from *Malva verticillata* seed via the activation of AMP-activated protein kinase. *Journal of Microbiology and Biotechnology*.

[2] Bowden L. G., Maini P. K., Moulton D. E. (2014). An ordinary differential equation model for full thickness wounds and the effects of diabetes. *Journal of Theoretical Biology*.

[3] Dos Santos M. S., Freitas M. N., de Oliveira Pinto F. (2014). O diabetes mellitus tipo 1 e tipo 2 e sua evolução no municipio de Quissamã-RJ. *Revista Científica Interdisciplinar*.

[4] Wilding J. P. H., Blonde L., Leiter L. A. (2015). Efficacy and safety of canagliflozin by baseline HbA1c and known duration of type 2 diabetes mellitus. *Journal of Diabetes and Its Complications*.

[5] Eddouks M., Bidi A., El Bouhali B., Hajji L., Zeggwagh N. A. (2014). Antidiabetic plants improving insulin sensitivity. *Journal of Pharmacy and Pharmacology*.

[6] International Diabetes Federation IDF https://www.idf.org/sites/default/files/EN_6E_Atlas_Full_0.pdf.

[7] International Diabetes Federation (IDF) (2011). *Diabetes Atlas*.

[8] Sociedade Brasileira de Endocrinologia e Metabologia (SBEM) http://www.endocrino.org.br/numeros-do-diabetes-no-brasil/.

[9] Ferreira V. A., Campos S. M. B. (2014). Avanços farmacológicos no tratamento do diabetes tipo 2. *Brazilian Journal of Surgery and Clinical Research*.

[10] Lawrence M. J., Rees G. D. (2000). Microemulsion-based media as novel drug delivery systems. *Advanced Drug Delivery Reviews*.

[11] Rosa R. L., Barcelos A. L. V., Bampi G. (2012). Investigação do uso de plantas medicinais no tratamento de indivíduos com diabetes melittus na cidade de Herval D' Oeste—SC. *Revista Brasileira de Plantas Medicinais*.

[12] Nain P., Saini V., Sharma S., Nain J. (2012). Antidiabetic and antioxidant potential of *Emblica officinalis* Gaertn. leaves extract in streptozotocin-induced type-2 diabetes mellitus (T2DM) rats. *Journal of Ethnopharmacology*.

[13] Abdel-Sattar E. A., Abdallah H. M., Khedr A., Abdel-Naim A. B., Shehata I. A. (2013). Antihyperglycemic activity of *Caralluma tuberculata* in streptozotocin-induced diabetic rats. *Food and Chemical Toxicology*.

[14] Petrovska B. B. (2012). Historical review of medicinal plants' usage. *Pharmacognosy Reviews*.

[15] Gadelha C. S. (2013). Estudo bibliográfico sobre o uso das plantas medicinais e fitoterápicos no Brasil. *Revista Verde de Agroecologia e Desenvolvimento Sustentável*.

[16] Feijó A. M., Bueno M. E., Ceolin T. (2012). Plantas medicinais utilizadas por idosos com diagnóstico de *Diabetes mellitus* no tratamento dos sintomas da doença. *Revista Brasileira de Plantas Medicinais*.

[17] Xu W., Zhou Q., Yin J.-J., Yao Y., Zhang J.-L. (2015). Anti-diabetic effects of polysaccharides from *Talinum triangulare* in streptozotocin (STZ)-induced type 2 diabetic male mice. *International Journal of Biological Macromolecules*.

[18] Schütz G. R., Sant'ana A. S. S., Santana S. G. (2011). Política de periódicos nacionais em Educação Física para estudos de revisão/sistemática. *Revista Brasileira de Cineantropometria & Desempenho Humano*.

[19] Moher D., Shamseer L., Clarke M. (2015). Preferred reporting items for systematic review and meta-analysis protocols (PRISMA-P) 2015 statement. *Systematic Reviews*.

[20] Pereira M. G., Galvão T. F. (2014). Etapas de busca e seleção de artigos em revisões sistemáticas da literatura. *Epidemiologia e Serviços de Saúde*.

[21] Hooijmans C. R., Leenaars M., Ritskes-Hoitinga M. (2010). A gold standard publication checklist to improve the quality of animal studies, to fully integrate the Three Rs, and to make systematic reviews more feasible. *Alternatives to Laboratory Animals*.

[22] Moher D., Liberati A., Tetzlaff J., Altman D. G. (2009). Preferred reporting items for systematic reviews and meta-analyses: the PRISMA statement. *Annals of Internal Medicine*.

[23] Schulz K. F., Altman D. G., Moher D. (2010). CONSORT 2010 statement: updated guidelines for reporting parallel group randomized trials. *Annals of Internal Medicine*.

[24] Kilkenny C., Browne W. J., Cuthill I. C., Emerson M., Altman D. G. (2013). Improving bioscience research reporting: the arrive guidelines for reporting animal research. *Animals*.

[25] Ren C., Zhang Y., Cui W. (2015). A polysaccharide extract of mulberry leaf ameliorates hepatic glucose metabolism and insulin signaling in rats with type 2 diabetes induced by high fat-diet and streptozotocin. *International Journal of Biological Macromolecules*.

[26] Miura T., Ichiki H., Hashimoto I. (2001). Antidiabetic activity of a xanthone compound, mangiferin. *Phytomedicine*.

[27] Kavishankar G. B., Lakshmidevi N. (2014). Anti-diabetic effect of a novel N-Trisaccharide isolated from *Cucumis prophetarum* on streptozotocin-nicotinamide induced type 2 diabetic rats. *Phytomedicine*.

[28] Moser C., Vickers S. P., Brammer R., Cheetham S. C., Drewe J. (2014). Antidiabetic effects of the *Cimicifuga racemosa* extract Ze 450 *in vitro* and *in vivo* in *ob/ob* mice. *Phytomedicine*.

[29] Ruan C.-T., Lam S.-H., Chi T.-C., Lee S.-S., Su M.-J. (2012). Borapetoside C from *Tinospora crispa* improves insulin sensitivity in diabetic mice. *Phytomedicine*.

[30] Sato M., Tai T., Nunoura Y., Yajima Y., Kawashima S., Tanaka K. (2002). Dehydrotrametenolic acid induces preadipocyte differentiation and sensitizes animal models of noninsulin-dependent diabetes mellitus to insulin. *Biological and Pharmaceutical Bulletin*.

[31] Lo H.-C., Tsai F.-A., Wasser S. P., Yang J.-G., Huang B.-M. (2006). Effects of ingested fruiting bodies, submerged culture biomass, and acidic polysaccharide glucuronoxylomannan of *Tremella mesenterica* Retz.:Fr. on glycemic responses in normal and diabetic rats. *Life Sciences*.

[32] Yoshida J., Seino H., Ito Y. (2013). Inhibition of glycogen synthase kinase-3*β* by falcarindiol isolated from Japanese parsley (*Oenanthe javanica*). *Journal of Agricultural and Food Chemistry*.

[33] Krenisky J. M., Luo J., Reed M. J., Carney J. R. (1999). Isolation and antihyperglycemic activity of bakuchiol from *Otholobium pubescens* (Fabaceae), a Peruvian medicinal plant used for the treatment of diabetes. *Biological and Pharmaceutical Bulletin*.

[34] Zhao R., Qiu B., Li Q. (2014). LBP-4a improves insulin resistance via translocation and activation of GLUT4 in OLETF rats. *Food and Function*.

[35] Perez-Gutierrez R. M., Damian-Guzman M. (2012). Meliacinolin: a potent *α*-glucosidase and *α*-amylase inhibitor isolated from *Azadirachta indica* leaves and in vivo antidiabetic property in streptozotocin-nicotinamide-induced type 2 diabetes in mice. *Biological and Pharmaceutical Bulletin*.

[36] Luo J., Cheung J., Yevich E. M. (1999). Novel terpenoid-type quinones isolated from *Pycnanthus angolensis* of potential utility in the treatment of type 2 diabetes. *Journal of Pharmacology and Experimental Therapeutics*.

[37] Kwon D. Y., Kim Y. S., Ryu S. Y. (2012). Platyconic acid, a saponin from *Platycodi radix*, improves glucose homeostasis by enhancing insulin sensitivity in vitro and in vivo. *European Journal of Nutrition*.

[38] Chen J., Ma M., Lu Y., Wang L., Wu C., Duan H. (2009). Rhaponticin from rhubarb rhizomes alleviates liver steatosis and improves blood glucose and lipid profiles in KK/Ay diabetic mice. *Planta Medica*.

[39] Hsu C.-Y., Shih H.-Y., Chia Y.-C. (2014). Rutin potentiates insulin receptor kinase to enhance insulin-dependent glucose transporter 4 translocation. *Molecular Nutrition and Food Research*.

[40] Kumar R., Patel D. K., Prasad S. K., Laloo D., Krishnamurthy S., Hemalatha S. (2012). Type 2 antidiabetic activity of bergenin from the roots of *Caesalpinia digyna* Rottler. *Fitoterapia*.

[41] Zhang Y., Wang J.-B., Wang L., Zhen L.-Y., Zhu Q.-Q., Chen X.-W. (2013). A study on hypoglycaemic health care function of *Stigma maydis* polysaccharides. *African Journal of Traditional, Complementary, and Alternative Medicines*.

[42] Agrawal R., Sethiya N. K., Mishra S. H. (2013). Antidiabetic activity of alkaloids of *Aerva lanata* roots on streptozotocin-nicotinamide induced type-II diabetes in rats. *Pharmaceutical Biology*.

[43] Paramaguru R., Mazumder P. M., Sasmal D., Jayaprakash V. (2014). Antidiabetic activity of *Pterospermum acerifolium* flowers and glucose uptake potential of bioactive fraction in L6 muscle cell lines with its HPLC fingerprint. *BioMed Research International*.

[44] Chakrabarti S., Biswas T. K., Seal T. (2005). Antidiabetic activity of *Caesalpinia bonducella* F. in chronic type 2 diabetic model in Long-Evans rats and evaluation of insulin secretagogue property of its fractions on isolated islets. *Journal of Ethnopharmacology*.

[45] Kiho T., Kochi M., Usui S., Hirano K., Aizawa K., Inakuma T. (2001). Antidiabetic effect of an acidic polysaccharide (TAP) from *Tremella aurantia* and its degradation product (TAP-H). *Biological and Pharmaceutical Bulletin*.

[46] Fujii M., Takei I., Umezawa K. (2009). Antidiabetic effect of orally administered conophylline-containing plant extract on streptozotocin-treated and Goto-Kakizaki rats. *Biomedicine & Pharmacotherapy*.

[47] Chen F., Xiong H., Wang J., Ding X., Shu G., Mei Z. (2013). Antidiabetic effect of total flavonoids from *Sanguis draxonis* in type 2 diabetic rats. *Journal of Ethnopharmacology*.

[48] Klomann S. D., Mueller A. S., Pallauf J., Krawinkel M. B. (2010). Antidiabetic effects of bitter gourd extracts in insulin-resistant db/db mice. *British Journal of Nutrition*.

[49] Arya A., Yeng Looi C., Chuen Cheah S., Rais Mustafa M., Ali Mohd M. (2012). Anti-diabetic effects of *Centratherum anthelminticum* seeds methanolic fraction on pancreatic cells, *β*-TC6 and its alleviating role in type 2 diabetic rats. *Journal of Ethnopharmacology*.

[50] Ibrahim M. A., Islam M. S. (2014). Anti-diabetic effects of the acetone fraction of *Senna singueana* stem bark in a type 2 diabetes rat model. *Journal of Ethnopharmacology*.

[51] Ganeshpurkar A., Kohli S., Rai G. (2014). Antidiabetic potential of polysaccharides from the white oyster culinary-medicinal mushroom *Pleurotus florida* (higher Basidiomycetes). *International Journal of Medicinal Mushrooms*.

[52] Shu X.-S., Lv J.-H., Tao J., Li G.-M., Li H.-D., Ma N. (2009). Antihyperglycemic effects of total flavonoids from *Polygonatum odoratum* in STZ and alloxan-induced diabetic rats. *Journal of Ethnopharmacology*.

[53] Roman-Ramos R., Almanza-Perez J. C., Fortis-Barrera A. (2012). Antioxidant and anti-inflammatory effects of a hypoglycemic fraction from *Cucurbita ficifolia* Bouché in streptozotocin-induced diabetes mice. *American Journal of Chinese Medicine*.

[54] Zhao R., Li Q., Xiao B. (2005). Effect of *Lycium barbarum* polysaccharide on the improvement of insulin resistance in NIDDM rats. *Yakugaku Zasshi*.

[55] Hwang I. K., Kim D. W., Park J. H. (2009). Effects of grape seed extract and its ethylacetate/ethanol fraction on blood glucose levels in a model of type 2 diabetes. *Phytotherapy Research*.

[56] Wu Y., Ou-Yang J.-P., Wu K., Wang Y., Zhou Y.-F., Wen C.-Y. (2005). Hypoglycemic effect of *Astragalus polysaccharide* and its effect on PTP1B. *Acta Pharmacologica Sinica*.

[57] Xu J., Wang Y., Xu D.-S., Ruan K.-F., Feng Y., Wang S. (2011). Hypoglycemic effects of MDG-1, a polysaccharide derived from *Ophiopogon japonicas*, in the ob/ob mouse model of type 2 diabetes mellitus. *International Journal of Biological Macromolecules*.

[58] Costantino L., Raimondi L., Pirisino R. (2003). Isolation and pharmacological activities of the *Tecoma stans* alkaloids. *Il Farmaco*.

[59] Bharti S. K., Kumar A., Sharma N. K. (2013). Tocopherol from seeds of *Cucurbita pepo* against diabetes: Validation by in vivo experiments supported by computational docking. *Journal of the Formosan Medical Association*.

[60] Kharbanda C., Sarwar Alam M., Hamid H. (2014). *Trapa natans* L. root extract suppresses hyperglycemic and hepatotoxic effects in STZ-induced diabetic rat model. *Journal of Ethnopharmacology*.

[61] Yu H., Zheng L., Xu L. (2015). Potent effects of the total saponins from *Dioscorea nipponica* Makino against streptozotocin-induced type 2 diabetes mellitus in rats. *Phytotherapy Research*.

[62] Zucker I., Beery A. K. (2010). Males still dominate animal studies. *Nature*.

[63] Machado C. C., Zatti R. A. (2013). Animais de laboratório: o camundongo. *Anais V SIMPAC*.

[64] Kilkenny C., Browne W. J., Cuthill I. C., Emerson M., Altman D. G. (2013). Improving bioscience research reporting: the ARRIVE guidelines for reporting animal research. *Animals*.

[65] Goud B. J., Dwarakanath V., Chikka B. K. (2015). Streptozotocin-a diabetogenic agent in animal models. *International Journal of Pharmacy & Pharmaceutical Research*.

[66] Furman B. L. (2015). Streptozotocin-induced diabetic models in mice and rats. *Current Protocols in Pharmacology*.

[67] Neto E. M. R., Marques L. A. R. V., Ferreira M. A. D. (2015). Metformin: a review of the literature. *Saúde e Pesquisa*.

[68] Shen S.-C., Chang W.-C. (2013). Hypotriglyceridemic and hypoglycemic effects of vescalagin from Pink wax apple [Syzygium samarangense (Blume) Merrill and Perry cv. Pink] in high-fructose diet-induced diabetic rats. *Food Chemistry*.

[69] Wang Y., Campbell T., Perry B., Beaurepaire C., Qin L. (2011). Hypoglycemic and insulin-sensitizing effects of berberine in high-fat diet- and streptozotocin-induced diabetic rats. *Metabolism: Clinical and Experimental*.

[70] Zheng T., Shu G., Yang Z., Mo S., Zhao Y., Mei Z. (2012). Antidiabetic effect of total saponins from *Entada phaseoloides* (L.) Merr. in type 2 diabetic rats. *Journal of Ethnopharmacology*.

[71] Negri G. (2005). Diabetes melito: plantas e princípios ativos naturais hipoglicemiantes. *Revista Brasileira de Ciências Farmacêuticas*.

[72] Al-Malki A. L. (2016). Inhibition of *α*-glucosidase by thiosulfinate as a target for glucose modulation in diabetic rats. *Evidence-Based Complementary and Alternative Medicine*.

[73] Wilson R. D., Islam M. S. (2012). Fructose-fed streptozotocin-injected rat: an alternative model for type 2 diabetes. *Pharmacological Reports*.

[74] Fonseca V. A. (2009). Defining and characterizing the progression of type 2 diabetes. *Diabetes Care*.

[75] Taha H., Arya A., Paydar M. (2014). Upregulation of insulin secretion and downregulation of pro-inflammatory cytokines, oxidative stress and hyperglycemia in STZ-nicotinamide-induced type 2 diabetic rats by Pseuduvaria monticola bark extract. *Food and Chemical Toxicology*.

[76] Silva A. D. S. E., Mota M. P. G. (2015). Efeitos dos programas de treinamento aeróbio, de força e combinado na glicose sanguínea em diabéticos do tipo 2: uma revisão sistemática. *Ciências em Saúde*.

[77] Kumar R., Pate D. K., Prasad S. K., Sairam K., Hemalatha S. (2011). Antidiabetic activity of alcoholic leaves extract of Alangium lamarckii Thwaites on streptozotocin-nicotinamide induced type 2 diabetic rats. *Asian Pacific Journal of Tropical Medicine*.

[78] Das D., Chaurasia A., Sahu P., Mishra V. K., Kashaw S. (2015). Antihypercholestrolemic potential of omega-3-fatty acid concentrate in alloxan induced diabetic rodent. *International Journal of Pharmaceutical Sciences and Research*.

[79] Pari L., Saravanan R. (2004). Antidiabetic effect of diasulin, a herbal drug, on blood glucose, plasma insulin and hepatic enzymes of glucose mretabolism hyperglycaemic rats. *Diabetes, Obesity and Metabolism*.

[80] Nagarajan N. S., Murugesh N., Thirupathy Kumaresan P., Radha N., Murali A. (2005). Antidiabetic and antihyperlipemic effects of *Clemeo felina*. *Fitoterapia*.

[81] Virdi N. S., Lefebvre P., Parisé H. (2013). Association of self-monitoring of blood glucose use on glycated hemoglobin and weight in newly diagnosed, insulin-naïve adult patients with type 2 diabetes. *Journal of Diabetes Science and Technology*.

